# The path of biomolecular mass spectrometry into open research

**DOI:** 10.1038/s41467-019-12150-4

**Published:** 2019-09-09

## Abstract

Originally designed for measuring isotope abundances and elemental masses, mass spectrometry is becoming a mainstay across life sciences. As electrospray ionization of biomolecules turns 30 and the Orbitrap mass analyzer 20, we take this opportunity to highlight the role of both inventions in stirring mass spectrometry from physics into biology and discuss the advances and challenges that may impact the future applications of biomolecular mass spectrometry.

The rate of scientific discovery is often dramatically accelerated by new methodological approaches or instruments. A few examples immediately come to mind: X-ray crystallography enabled the visualization of proteins in three-dimensional space; massively parallel sequencing transformed the fields of genomics, transcriptomics and epigenetics by increasing throughput and decreasing sequencing costs by orders of magnitude; and CRISPR-Cas now allows genetic manipulations to be carried out with a level of speed and accuracy that would have qualified as science fiction a mere 10 years ago. 2019 marks the decennial anniversaries of two such innovations in the application of mass spectrometry that have transformed several fields of biological and clinical research: electrospray ionization of large biomolecules, and their high-resolution detection with the Orbitrap mass analyzer.

Electrospray ionization (ESI) was originally described in 1984 by the 2002 Nobel laureate John B. Fenn and his colleague Masamichi Yamashita^1^; who showed that inserting an aqueous sample solution in a conductive capillary and applying an electrical current allows generating gaseous ions of the sample compounds that can be measured by mass spectrometry. Following the demonstration that ESI enables the mass spectrometric analysis of labile biomolecules, Fenn and his coworkers brought ESI-mass spectrometry into the biology limelight with a review article published in 1989^2^. Ten years later, Alexander Makarov introduced the Orbitrap mass analyzer, presenting a new approach enabling robust mass measurements at high resolution^3^. Although it was not until 2005 that a commercial Orbitrap mass spectrometer became available, Makarov’s presentation in 1999 provided the first glimpse into the underlying principles of what is today one of the most widely used mass analyzers for proteomics and analytical chemistry.

To highlight the anniversaries of these two key enabling advances in the life sciences, we recently unveiled a collection of articles published in *Nature Communications* that showcases applications of ESI- and Orbitrap-mass spectrometry in the life and clinical sciences. The collection features recent research, review and perspective articles, as well as papers that have attracted our readers’ attention over several years. It covers proteomics, approaches to localize and quantify post-translational modifications, mass spectrometry-based structural biology, and mass spectrometric analyses of other biomolecules such as carbohydrates, nucleic acids and metabolites. In two historical perspectives, the Orbitrap inventor Alexander Makarov recounts how this mass analyzer came into being and Matthias Mann, John Fenn’s graduate student and one of the first researchers to analyze proteins by mass spectrometry^2^, revisits the invention of ESI and the subsequent emergence of proteomics^4,5^.

Notwithstanding the importance of ESI and the Orbitrap analyzer, there are many other influential developments that paved the way towards biological mass spectrometry (www.nature.com/milestones/mass-spec). As both Mann^5^ and Makarov^4^ point out, key elements of state-of-the art mass spectrometers were invented long before the application of mass spectrometry to biomolecules. Following the works of the physicists Joseph John (J.J.) Thomson and Wilhelm Wien on positive rays and cathode rays^6,7^, mass spectrometry arose as an analytical technique that was first applied to measuring stable elemental isotopes in the gas phase^8^. Owing to the development of additional methods to ionize and fragment analytes, new mass analyzers to measure their mass-to-charge ratios, and increasingly comprehensive compound libraries, mass spectrometry soon became an established tool in chemical research. However, mass spectrometric analysis of proteins and nucleic acids remained out of reach because their more labile peptide or sugar-phosphate backbones were lost during the transfer into the gas phase. This changed— allowing mass spectrometry to enter the realm of biology— with the invention of ESI^2^, where charged droplets of sample solution gradually desolvate until only ‘naked’ molecular ions remain; and matrix-assisted laser desorption/ionization (MALDI)^9,10^, which relies on embedding biomolecules in an energy-absorbing crystalline matrix ionized with a laser pulse of appropriate energy and wavelength.

While the majority of biological mass spectrometry applications today rely on ESI, MALDI remains widely used to visualize the spatial distribution of molecules in histological mass spectrometry imaging experiments^11^ and for the rapid identification of microorganisms in clinical microbiology^12^. These and other recent developments^13^ illustrate how the application of mass spectrometry has expanded from physics into chemistry, biology and even clinical research. Being relevant to so many disciplines, mass spectrometry based research finds a natural home within *Nature Communications*. A study applying mass spectrometry to discover a protein methyltransferase was published within our first 50 articles^14^ and we have since then been striving to publish significant advances in all areas of mass spectrometry.

Since *Nature Communications’* first mass spectrometry paper, the field has continued to evolve. Mass spectrometers and data analysis tools have become substantially more powerful and accessible to researchers across life and clinical sciences – in part because new generations of Orbitraps and other ESI-based instruments have been made commercially available. Operating these instruments has become increasingly intuitive and less dependent on in-depth technical expertise in mass spectrometry. While these technological advances offer exciting opportunities, they also raise expectations. At *Nature Communications*, we expect biological and clinical studies that use mass spectrometry to thoroughly validate discovery phase results and demonstrate their physiological relevance. Similarly, studies describing new or improved mass spectrometry methods and workflows must stand up to rigorous benchmarking against existing approaches and provide compelling proof-of-concept applications. Concomitantly, as the demands for data and method transparency continue to increase, all areas of mass spectrometry applications are challenged with regard to standardization of analytical workflows and data sharing. Mass spectrometry users have made significant headway in addressing these issues by proposing best practice guidelines for various disciplines and by improving the online data repositories, yet several hurdles remain. Reproducible protocols and standardized data reporting—still not universally embraced in fundamental biological mass spectrometry—become essential when analyzing and comparing large cohorts of samples. Depositing mass spectral raw data to publically accessible repositories remains to become the norm outside of proteomics, which is partially attributable to the fact that suitable community repositories are still lacking. Beyond expanding standardized raw data repositories, mass spectrometry-based clinical research calls for data sharing workflows that provide access to the data without compromising patient confidentiality and without requiring reviewers and other researchers to reveal their identity to the study lead.

“… as the demands for data and method transparency continue to increase, all areas of mass spectrometry applications are challenged with regard to standardization of analytical workflows and data sharing.”

For both new applications and methodological advances in mass spectrometry, it is increasingly clear that their full potential will be realized only if methods, data, code, and software are reported in accordance with the community guidelines and made findable, accessible, interoperable, and reusable (FAIR^15^). On its way to becoming a FAIR research field, biomolecular mass spectrometry must overcome the obstacles outlined above, which will require concerted efforts from all parties involved in the scientific process. This presents a formidable challenge, but it is well worth the effort. As 2019 also marks the fifth anniversaries of the human proteome drafts—two landmark projects that critically relied on open data^16,17^—the jubilees of 2019 remind us that bringing mass spectrometry forward requires both ingenious concepts and public sharing of the amazing science they spawn.
